# Mental health of migrants during the COVID-19 pandemic: additional stressors, increased inequalities

**DOI:** 10.1093/eurpub/ckac131.501

**Published:** 2022-10-25

**Authors:** MJ Marques, A Gama, AR Pedro, R Magano, C Tiessen, P Hollanders, S Dias

**Affiliations:** 1 NOVA National School of Public Health, Universidade NOVA de Lisboa, Lisbon, Portugal; 2 Comprehensive Health Research Center, Universidade NOVA de Lisboa, Lisbon, Portugal; 3 Vrije Universiteit Amsterdam, Amsterdam, Netherlands; 4 Maastricht University, Maastricht, Netherlands

## Abstract

The COVID-19 pandemic has resulted in an unprecedent range of negative mental health outcomes across populations worldwide. Such effects are increasingly being documented, however an evidence gap persists on the consequences on most vulnerable groups, as certain subgroups of migrants. These populations already suffer from increased psychological burden, and the pandemic effects may potentially exacerbate adverse experiences and outcomes. This study aims to uncover the perceived impact of the COVID-19 pandemic on the mental health of migrants in Portugal and the associated sociodemographic aspects. A survey was conducted with a community-based sample of 1126 adult migrants in Portugal, assessing sociodemographics, migration-related characteristics and the perceived impact of the pandemic on mental health. Association between sociodemographics and mental health indicators was measured through bivariable analysis. In total, 1126 adult migrants were surveyed: 53.4% female, mean age of 35.8 years (range 18-77), 48.9% from African countries, 29.5% from Middle East/Asian countries, 21.6% from Brazil. Most participants (80%) reported feelings of agitation, anxiety or sadness during the pandemic period with 26.4% experiencing these feelings most days. The pandemic had a disproportionate impact on women (86.9% reported negative impact compared to 72.5% of men, p < 0.05), those undocumented (83.3% vs 75.4%, p < 0.05), those whose financial situation got worse since the pandemic (82.8% vs. 77.3%, p < 0.05) and those who had increased food shortages (84.4% vs 79%, p < 0.05). Migrants perceived an elevated deterioration of their mental health during the COVID-19 pandemic. In addition, particular groups such as women and those with a more insecure income or residence status are particularly susceptible to experiencing negative mental health outcomes.

**Key messages:**

• There is a need to recognize the detrimental mental health impact of the COVID-19 pandemic on particular migrant groups and to develop interventions that target their unique needs.

• Investigating sociodemographic and migration aspects could help identifying migrants at a higher risk of experiencing mental health distress.

